# Genomic prediction powered by multi-omics data

**DOI:** 10.3389/fgene.2025.1636438

**Published:** 2025-09-17

**Authors:** Osval A. Montesinos-López, Abelardo Montesinos-López, Brandon Alejandro Mosqueda-González, Iván Delgado-Enciso, Moises Chavira-Flores, José Crossa, Susanne Dreisigacker, Jin Sun, Rodomiro Ortiz

**Affiliations:** ^1^ Facultad de Telemática, Universidad de Colima, Colima, Mexico; ^2^ Centro Universitario de Ciencias Exactas e Ingenierías (CUCEI), Universidad de Guadalajara, Guadalajara, Jalisco, Mexico; ^3^ Institut National des Sciences Appliquées de Lyon, Villeurbanne, France; ^4^ School of Medicine, University of Colima, Colima, Mexico; ^5^ Instituto de Investigaciones en Matemáticas Aplicadas y Sistemas (IIMAS), Universidad Nacional Autónoma de México (UNAM), Mexico City, Mexico; ^6^ Colegio de Postgraduados (COLPOS), Montecillos, Mexico; ^7^ International Maize and Wheat Improvement Center (CIMMYT), Mexico; ^8^ Department of Statistics, School of Science, Yanshan University, Qinhuangdao, China; ^9^ Department of Plant Breeding at SLU, Swedish University of Agricultural Sciences, Uppsala, Sweden

**Keywords:** genomic selection, omics data, optimal integration, plant breeding, prediction accuracy

## Abstract

Genomic selection (GS) has transformed plant breeding by enabling early and accurate prediction of complex traits. However, its predictive performance is often constrained by the limited information captured through genomic markers alone, especially for traits influenced by intricate biological pathways. To address this, the integration of complementary omics layers—such as transcriptomics and metabolomics—has emerged as a promising strategy to enhance prediction accuracy by providing a more comprehensive view of the molecular mechanisms underlying phenotypic variation. We used three datasets, each collected under a single-environment condition, which allowed us to isolate the effects of omics integration without the confounding influence of genotype-by-environment interaction. We assessed 24 integration strategies combining three omics layers: genomics, transcriptomics, and metabolomics. These strategies encompassed both early data fusion (concatenation) and model-based integration techniques capable of capturing non-additive, nonlinear, and hierarchical interactions across omics layers. The evaluation was conducted using three real-world datasets from maize and rice, which varied in population size, trait complexity, and omics dimensionality. Our results indicate that specific integration methods—particularly those leveraging model-based fusion—consistently improve predictive accuracy over genomic-only models, especially for complex traits. Conversely, several commonly used concatenation approaches did not yield consistent benefits and, in some cases, underperformed. These findings underscore the importance of selecting appropriate integration strategies and suggest that more sophisticated modeling frameworks are necessary to fully exploit the potential of multi-omics data. Overall, this work highlights both the value and limitations of multi-omics integration for genomic prediction and offers practical insights into the design of omics-informed selection strategies for accelerating genetic gain in plant breeding programs.

## Introduction

Genomic selection (GS) has revolutionized the field of plant breeding by enabling the selection of superior genotypes based on genomic estimated breeding values (GEBVs) derived from dense molecular marker information. Initially proposed by Meuwissen et al. (2001), GS bypasses the need for direct phenotypic selection, allowing for early and more efficient selection decisions, thereby shortening the breeding cycle and enhancing genetic gain. This methodology represents a fundamental shift in the breeder’s toolbox, moving from phenotype-based to genotype-driven decision-making, and has been successfully implemented in numerous crop breeding programs worldwide (Crossa et al., 2017; Desta and Ortiz, 2014).

Despite its transformative potential, the implementation of GS in real-world breeding programs faces several challenges. One key limitation is the variability in prediction accuracy across different environments and breeding populations. Factors such as genotype-by-environment $\left(G\times E\right)$ interactions, limited training population sizes, and the genetic architecture of traits of interest can significantly hinder the robustness of genomic predictions (Roorkiwal et al., 2016). Additionally, the cost and logistics of genotyping and phenotyping large populations remain substantial obstacles, particularly in resource-limited settings. These challenges necessitate the development of novel strategies to optimize the accuracy and applicability of GS.

In response to these challenges, a growing body of research has focused on improving the prediction accuracy of GS models. Strategies such as optimizing training population design (Rincent et al., 2012), incorporating G×E interactions (Jarquín et al., 2014), and applying advanced statistical learning techniques (Montesinos-López et al., 2018) have shown promising results. However, even with these improvements, the integration of additional layers of biological data offers a compelling avenue for further enhancing model performance.

The integration of multi-omics data—including genomics, transcriptomics, metabolomics, and proteomics—has emerged as a powerful strategy to enhance the performance of genomic prediction (GP) models in plant and animal breeding. These diverse yet complementary datasets provide a multidimensional view of the complex biological systems that govern phenotypic expression, enabling a more precise dissection of the genotype-to-phenotype relationship. Unlike single-layer genomic data, which often capture only a portion of the heritable variance, multi-omics approaches can account for regulatory, transcriptional, post-transcriptional, and metabolic interactions that influence trait architecture. For example, transcriptomic data capture gene expression levels across tissues or developmental stages, shedding light on functional genes and regulatory networks underlying complex traits (Guo et al., 2016; Azodi et al., 2020). Similarly, metabolomic profiles offer dynamic snapshots of cellular biochemical processes, which are often directly associated with phenotypic traits such as growth, stress response, or yield (Riedelsheimer et al., 2012; Wen et al., 2014). Proteomics data, although less frequently used due to technical constraints, provide critical insights into post-translational modifications and protein abundance, which are closely tied to phenotypic outcomes (Misra et al., 2019). The synergistic integration of these omics layers can substantially improve the explanatory power of prediction models, particularly for complex traits governed by multiple small-effect loci and their downstream interactions (Wang M. et al., 2024). Furthermore, machine learning and statistical modeling techniques have increasingly enabled the effective fusion of high-dimensional omics data into genomic selection pipelines, resulting in significant gains in predictive accuracy (Montesinos-López et al., 2022). As such, multi-omics integration not only enriches the biological relevance of genomic predictions but also facilitates more informed decision-making in breeding programs aimed at improving crop resilience, productivity, and nutritional quality.

Several studies have demonstrated the utility of integrating multi-omics data into GS models. For example, Azodi et al. (2020) showed that combining gene expression data with genomic information improved the prediction of complex traits in maize. Similarly, Riedelsheimer et al. (2012) found that metabolite profiles significantly contributed to the prediction of biomass traits in maize hybrids.

Wang et al. (2024b) developed an extensive multi-omics atlas for wheat, integrating transcriptomic, proteomic, phosphoproteomic, and acetylproteomic data across various tissues and developmental stages. This integrative approach enhanced the understanding of complex traits, including disease resistance and grain quality, highlighting the potential of multi-omics data to improve the predictive accuracy of genomic selection models.

Despite its potential, the statistical integration of heterogeneous omics datasets presents significant challenges. These arise from inherent differences in data dimensionality, measurement scales, noise levels, and patterns of missingness across various omics platforms. Additionally, capturing the intricate—and often nonlinear—interactions both within and between omics layers, and their combined influence on complex phenotypes, requires highly sophisticated analytical frameworks. Traditional linear models commonly employed in GS may lack the flexibility to adequately model these multidimensional relationships. Consequently, there is an increasing need to adopt advanced machine learning approaches, including deep learning architectures, kernel-based methods, and Bayesian hierarchical models, which offer greater adaptability and capacity to uncover hidden structures in complex biological data (Montesinos-López et al., 2019; Montesinos-López et al., 2022).

Another critical aspect is the model tuning process. Although machine learning approaches are often highly competitive compared to traditional methods in predictive accuracy, they are frequently associated with complex and computationally intensive tuning procedures. This complexity can limit their practical applicability, especially in high-dimensional omics contexts. As such, the development of methodologies that strike a balance between predictive performance and user-friendly tuning remains a pressing research priority. Furthermore, the standardization of data preprocessing pipelines and the assurance of data quality across omics layers are essential for enhancing the reliability and reproducibility of integrative analyses.

Several recent studies have explored multi-omics integration using deep learning to predict phenotypic traits in crops and model species (Angermueller et al., 2016; Zingaretti et al., 2020; Montesinos-López et al., 2021). Although promising, most approaches have been limited by dataset size, environmental heterogeneity, or lack of benchmarking across model types. Our study addresses these limitations by evaluating predictive performance across three distinct datasets using standardized cross-validation procedures and multiple deep learning architectures.

In this research, we aim to address these challenges by integrating genomic, transcriptomic, and metabolomic data to explore alternative modeling approaches for improving GS methodology. The availability of these datasets enables the application of statistical and machine learning methods to predict complex traits by integrating genomic (*G*), transcriptomic (*T*), and metabolomic (*M*) data. By leveraging the complementary information provided by each omics layer, our goal is to enhance the prediction of complex agronomic traits in plant breeding. This integrative framework not only holds promise for improving model accuracy but also offers a deeper understanding of the biological mechanisms driving trait variation. We first evaluate how omics-based similarity among these datasets relates to trait variation.

Our study contributes to the growing body of evidence supporting multi-omics integration in GS and aims to identify modeling strategies that effectively harness the rich biological information embedded in diverse omics datasets. We explore conventional statistical learning methodologies capable of addressing the unique challenges of multi-omics integration, with an emphasis on practical implementation in plant breeding programs. Ultimately, this research aims to provide breeders with more accurate and biologically informed tools to accelerate genetic improvement.

## Materials and methods

### Datasets

We used three datasets previously presented by Yang et al. (2022) for benchmarking the proposed predictors. These datasets were collected under a single-environment condition and contain various continuous traits along with metabolomic and transcriptomic data. Table 1 summarizes the characteristics of three multi-omics datasets used in this research. The Maize282 dataset includes 279 lines evaluated for 22 phenotypic traits, with high-density genotypic data comprising 50,878 markers, along with 18,635 metabolomic and 17,479 transcriptomic features. The Maize368 dataset consists of 368 lines assessed for 20 traits, with a larger genotypic matrix of 100,000 markers, complemented by 748 metabolomic and 28,769 transcriptomic variables. The Rice210 dataset comprises 210 lines evaluated for four traits, with comparatively fewer genotypic markers (1,619) and metabolomic features (1,000), but a similarly large transcriptomic profile of 24,994 features.

**TABLE 1:** Summary of the three datasets used in the study.

Dataset	Lines	Traits	Markers	Metabolomics	Transcriptomics
Maize282	279	22	50,878	18,635	17,479
Maize368	368	20	100,000	748	28,769
Rice210	210	4	1,619	1,000	24,994

These datasets illustrate the diversity in sample size, trait complexity, and omics layer dimensionality across species, highlighting the analytical challenges in integrative modeling. More details about these datasets can be found at the following link: https://doi.org/10.6084/m9.figshare.19312205.v1.

### Statistical models

#### Model 1 (M1)

The basic Bayesian genomic best linear unbiased predictor (GBLUP) model incorporates only genomic main effects using the genomic relationship matrix (**G**) and is defined as follows:

Y=1μ+P+ϵ. (1)

Here, Y represents the vector of the continuous response variable observed of order n×1 . 1 denotes a vector of ones of order n×1 . μ stands for the general mean or intercept. $P={\left(P_{1},\ldots ,P_{n}\right)}^{T}$ denotes a predictor that contains at least one random effect associated with the vector of genotypes. In the case of model M1 the predictor (P) contains only the genomic information. Additionally, ϵ denotes the vector random error components for vector of genotypes, where each error is independently and normally distributed with a mean of 0 and a variance of $\sigma^{2}=Ve$ . All predictors implemented are provided in Table 2.

**TABLE 2:** Evaluated models. Here, each model corresponds to a different predictor.

Model	Predictor (P)
M1	$g_{L}$
M2	$t_{L}$
M3	$m_{L}$
M4	$g_{L}+t_{L}+m_{L}$
M5	$g_{L}+t_{L}+m_{L}+{\left(g_{L}t_{L}\right)}^{CC}+{\left(g_{L}t_{L}\right)}^{PP}$
M6	$g_{L}+t_{L}+m_{L}+{\left(g_{L}m_{L}\right)}^{CC}+{\left(g_{L}m_{L}\right)}^{PP}$
M7	$g_{L}+t_{L}+m_{L}+{\left(t_{L}m_{L}\right)}^{CC}+{\left(t_{L}m_{L}\right)}^{PP}$
M8	$g_{L}+t_{L}+m_{L}+{\left(g_{L}t_{L}\right)}^{CC}+{\left(g_{L}t_{L}\right)}^{PP}+{\left(g_{L}m_{L}\right)}^{CC}+{\left(g_{L}m_{L}\right)}^{PP}$
M9	$g_{L}+t_{L}+m_{L}+{\left(g_{L}t_{L}\right)}^{CC}+{\left(g_{L}t_{L}\right)}^{PP}+{\left(t_{L}m_{L}\right)}^{CC}+{\left(t_{L}m_{L}\right)}^{PP}$
M10	$g_{L}+t_{L}+m_{L}+{\left(g_{L}m_{L}\right)}^{CC}+{\left(g_{L}m_{L}\right)}^{PP}+{\left(t_{L}m_{L}\right)}^{CC}+{\left(t_{L}m_{L}\right)}^{PP}$
M11	$g_{L}+t_{L}+m_{L}+{\left(g_{L}t_{L}\right)}^{CC}+{\left(g_{L}t_{L}\right)}^{PP}+{\left(g_{L}m_{L}\right)}^{CC}+{\left(g_{L}m_{L}\right)}^{PP}+{\left(t_{L}m_{L}\right)}^{CC}+{\left(t_{L}m_{L}\right)}^{PP}$
M12	$g_{G}$
M13	$t_{G}$
M14	$m_{G}$
M15	$g_{G}+t_{G}+m_{G}$
M16	$g_{G}+t_{G}+m_{G}+{\left(g_{G}t_{G}\right)}^{CC}+{\left(g_{G}t_{G}\right)}^{PP}$
M17	$g_{G}+t_{G}+m_{G}+{\left(g_{G}m_{G}\right)}^{CC}+{\left(g_{G}m_{G}\right)}^{PP}$
M18	$g_{G}+t_{G}+m_{G}+{\left(t_{G}m_{G}\right)}^{CC}+{\left(t_{G}m_{G}\right)}^{PP}$
M19	$g_{G}+t_{G}+m_{G}+{\left(g_{G}t_{G}\right)}^{CC}+{\left(g_{G}t_{G}\right)}^{PP}+{\left(g_{G}m_{G}\right)}^{CC}+{\left(g_{G}m_{G}\right)}^{PP}$
M20	$g_{G}+t_{G}+m_{G}+{\left(g_{G}t_{G}\right)}^{CC}+{\left(g_{G}t_{G}\right)}^{PP}+{\left(t_{G}m_{G}\right)}^{CC}+{\left(t_{G}m_{G}\right)}^{PP}$
M21	$g_{G}+t_{G}+m_{G}+{\left(g_{G}m_{G}\right)}^{CC}+{\left(g_{G}m_{G}\right)}^{PP}+{\left(t_{G}m_{G}\right)}^{CC}+{\left(t_{G}m_{G}\right)}^{PP}$
M22	$g_{G}+t_{G}+m_{G}+{\left(g_{G}t_{G}\right)}^{CC}+{\left(g_{G}t_{G}\right)}^{PP}+{\left(g_{G}m_{G}\right)}^{CC}+{\left(g_{G}m_{G}\right)}^{PP}+{\left(t_{G}m_{G}\right)}^{CC}+{\left(t_{G}m_{G}\right)}^{PP}$
M23	$g_{L}+t_{L}+m_{L}+g_{G}+t_{G}+m_{G}$
M24	$g_{L}+t_{L}+m_{L}+g_{G}+t_{G}+m_{G}+{\left(g_{G}t_{G}\right)}^{CC}+{\left(g_{G}t_{G}\right)}^{PP}+{\left(g_{G}m_{G}\right)}^{CC}+{\left(g_{G}m_{G}\right)}^{PP}+{\left(t_{G}m_{G}\right)}^{CC}+{\left(t_{G}m_{G}\right)}^{PP}$

### Predictors

All predictors evaluated (Equation 1) varied depending on the source of omics data used. For this reason, the predictors (given in Equation 1) comprise different combinations of markers ( G ), metabolomic ( M ) and transcriptomic ( T ) data, each evaluated with two different kernel functions: linear, denoted with L subscript, and Gaussian, denoted with G subscript. The integration of omics datasets was performed using early data fusion: after normalization and mean imputation, all block features from the genomic, transcriptomic, and metabolomic layers and combinations of these feature layers were modeled as separate random effects under a mixed-model framework. This modeling framework has the advantage of correctly partitioning variance among different sources (genomic, transcriptomic, metabolomic, etc.) and leads to more accurate and unbiased estimates of fixed effects. It also improves the prediction accuracy of random components, such as breeding values in plant and animal genetics. Although this approach facilitates unified modeling, it introduces limitations such as computational intensity, noise accumulation, and increased risk of overfitting due to the high number of predictors in the input datasets.

#### Model 1 (M1)

For example, when ${P=g}_{L}$ , the model denoted as M1 represents a random effect with genomic information in terms of a linear kernel. It is assumed that $g_{L}∼{\left(g_{1},\ldots ,g_{n}\right)}^{T}∼N_{J}\left(0,\sigma_{g}^{2}K_{g}\right)$ , where $\sigma_{g}^{2}$ is the variance component for lines using genomic information and $K_{g}$ is a linear kernel referred to as the genomic relationship matrix, calculated using the method outlined by VanRaden (2008).

#### Models 2 and 3 (M2 and M3)

In a similar fashion, when ${P=t}_{L}$ , which is called model 2 (M2), this predictor represents a random effect of lines with the transcriptomic information in terms of a linear kernel. That is, $t_{L}∼{\left(t_{1},\ldots ,t_{n}\right)}^{T}∼N_{n}\left(0,\sigma_{t}^{2}K_{t}\right)$ , where $\sigma_{t}^{2}$ is the variance component for lines using transcriptomic information and $K_{t}$ is a linear transcriptomic relationship matrix. Furthermore, when ${P=m}_{L}$ , the model is called model M3; this predictor represents a random effect of lines with the metabolomic information in terms of a linear kernel. That is, $m_{L}∼{\left(m_{1},\ldots ,m_{n}\right)}^{T}∼N_{n}\left(0,\sigma_{m}^{2}K_{m}\right)$ , where $\sigma_{m}^{2}$ is the variance component for lines using metabolomic information and $K_{m}$ is a linear metabolomic relationship matrix. Thus, **M3** integrates transcriptomic data using kernel $K_{t}$ .

#### Models 4 and 5 (M4 and M5)

Model 4 (M4) is a predictor that includes the three previous random effects, that is, $P{={g_{L}+t}_{L}+m}_{L}.$ Model M5 contains the predictor $P=g_{L}+t_{L}+m_{L}+{\left(g_{L}t_{L}\right)}^{CC}+{\left(g_{L}t_{L}\right)}^{PP}$ . That is, it is equivalent to model M4 plus two additional terms. The first term in this predictor is distributed as ${\left(g_{L}t_{L}\right)}^{CC}∼N_{n}\left(0,\sigma_{gt}^{2}K_{gt}^{CC}\right)$ , where $\sigma_{gt}^{2}$ is the variance component for lines using transcriptomic and genomic information and $K_{gt}^{CC}$ is computed using the upper triangular (**UT**) part of the matrix resulting from the multiplication of the linear kernels $K_{g}$ and $K_{t}$ , that is, $K_{gt}^{CC}=$ **UT** + t (**UT**) (Cuevas, et al., 2025). On the other hand, the second term in this predictor is distributed as ${\left(g_{L}t_{L}\right)}^{PP}∼N_{n}\left(0,\sigma_{gtp}^{2}K_{gt}^{PP}\right)$ , where $\sigma_{gtp}^{2}$ is the variance component for lines using transcriptomic and genomic information and $K_{gt}^{PP}$ is computed using the lower triangular (**LT**) part of the same matrix multiplication of the linear kernels $K_{g}$ and $K_{t}$ , that is, $K_{gt}^{PP}=$ **LT** + t (**LT**) (Cuevas, et al., 2025).

In this case, the $K_{gt}^{CC}$ and $K_{gt}^{PP}$ kernels capture complex interactions between genomic and transcriptomic information that are not accounted for by the traditional interaction term based on the Hadamard product of the $K_{g}$ and $K_{t}$ kernels. Moreover, these new kernels are valid because they satisfy the three essential conditions for a kernel: (1) symmetry, (2) positive semi-definiteness (PSD), and (3) Mercer’s condition for continuous kernels.

#### Models 5–7 (M5–M7)

In the predictor of model M6, the first three terms are equal to those of model M5, but the remaining two are different; these last two terms are distributed as ${\left(g_{L}m_{L}\right)}^{CC}∼N_{n}\left(0,\sigma_{gm}^{2}K_{gm}^{CC}\right)$ and ${\left(g_{L}m_{L}\right)}^{PP}∼N_{n}\left(0,\sigma_{gmp}^{2}K_{gm}^{PP}\right)$ , where $\sigma_{gm}^{2}$ and $\sigma_{gmp}^{2}$ are variance components for lines using genomic and metabolomic information and $K_{gm}^{CC}$ and $K_{gm}^{PP}$ were computed in the same way as in model M5, but here the **UT** and **LT** parts are taken from the multiplication of the linear kernels $K_{g}$ and $K_{m}$ (Cuevas, et al., 2025). Furthermore, model M7 is equivalent to model M5, except that the last two terms differ. In M7, these terms are distributed as ${\left(t_{L}m_{L}\right)}^{CC}∼N_{n}\left(0,\sigma_{tm}^{2}K_{tm}^{CC}\right)$ and ${\left(t_{L}m_{L}\right)}^{PP}∼N_{n}\left(0,\sigma_{tmp}^{2}K_{tm}^{PP}\right)$ , where $\sigma_{tm}^{2}$ and $\sigma_{tmp}^{2}$ are variance components for lines using transcriptomic and metabolomic information and $K_{tm}^{CC}$ and $K_{tm}^{PP}$ were computed in the same way as in model M5, but here the **UT** and **LT** parts are taken from the multiplication of the linear kernels $K_{t}$ and $K_{m}$ (Cuevas, et al., 2025).

Note that the matrices $K_{t}$ and $K_{m}$ were computed as Gaussian kernel matrices from the transcriptomic and metabolomic data, respectively, using Euclidean distance and a default bandwidth parameter of *σ* = median (d^2^), where d^2^ denotes squared distances among individuals. As previously mentioned, to capture nonlinear interactions between transcriptomic and metabolomics effects, we decomposed the matrix multiplication of $K_{t}$ and $K_{m}$ into two components: the **UT** matrix and the **LT** matrix. These components capture complex interactions that are not accounted for by the conventional interaction term based on the Hadamard product of the $K_{t}$ and $K_{m}$ kernels. This approach aims to reflect distinct transcriptional and metabolite mechanisms across the distribution and improve the biological relevance of interaction modeling.

#### Models 8–14 (M8–M14)

The predictor of M8 is equivalent to that of M5 plus the last two terms of M6, while the predictor of model M9 is equivalent to that of M5 plus the last two terms of M7. The predictor of M10 is equivalent to that of M6 plus the last two terms of M7, while the predictor of M11 is equivalent to that of M10 plus the last two terms of model M5. On the other hand, model (M12) is equivalent to model M1, except that a Gaussian Kernel is used in place of a linear kernel. In this case, ${P=g}_{G}$ and $g_{G}∼{\left(g_{1},\ldots ,g_{n}\right)}^{T}∼N_{J}\left(0,\sigma_{Gg}^{2}K_{Gg}\right)$ , where $K_{Gg}$ is a Gaussian kernel computed using marker information. Similarly, model (M13) is equivalent to model M2 but replaces the linear kernel with a Gaussian Kernel. In this case, ${P=t}_{G}$ and $t_{G}∼{\left(t_{1},\ldots ,t_{n}\right)}^{T}∼N_{n}\left(0,\sigma_{Gt}^{2}K_{Gt}\right)$ , where $K_{Gt}$ is a Gaussian kernel computed using transcriptomic information. Similarly, model (M14) is equivalent to model M3 but uses a Gaussian Kernel $m_{G}∼{\left(m_{1},\ldots ,m_{n}\right)}^{T}∼N_{n}\left(0,\sigma_{Gm}^{2}K_{Gm}\right)$ , where $K_{Gm}$ is a Gaussian kernel computed using metabolomic information.

#### Models 15–24 (M15 and M24)

Model M15 is equivalent to model M4 but replaces the linear kernels with the corresponding Gaussian kernels. Model M16 is equivalent to model M5 but replaces the linear kernels with the corresponding Gaussian kernels. Model M17 is equivalent to model M6 but replaces the linear kernels with the corresponding Gaussian kernels. Model M18 is equivalent to model M7 but replaces the linear kernels with the corresponding Gaussian kernels. In a similar fashion, models M19, M20, M21, and M22 are equivalent to models M8, M9, M10 and M11, respectively, but replace the linear kernels with the corresponding Gaussian kernels. On the other hand, model M23 combines the terms of models M4 and M15. Finally, model M24 is equivalent to model M22 plus the terms of model M15. More details of these 24 models are provided in Table 2. In addition, we use the CC and PP superscripts to refer to those terms in each model that contain hybrid kernels derived from the product of two linear or Gaussian kernels. All these models were implemented in R statistical software (R Core Team, 2025) utilizing the BGLR library (Pérez and de los Campos, 2014).

### Model’s relationships

**FIGURE 1:**
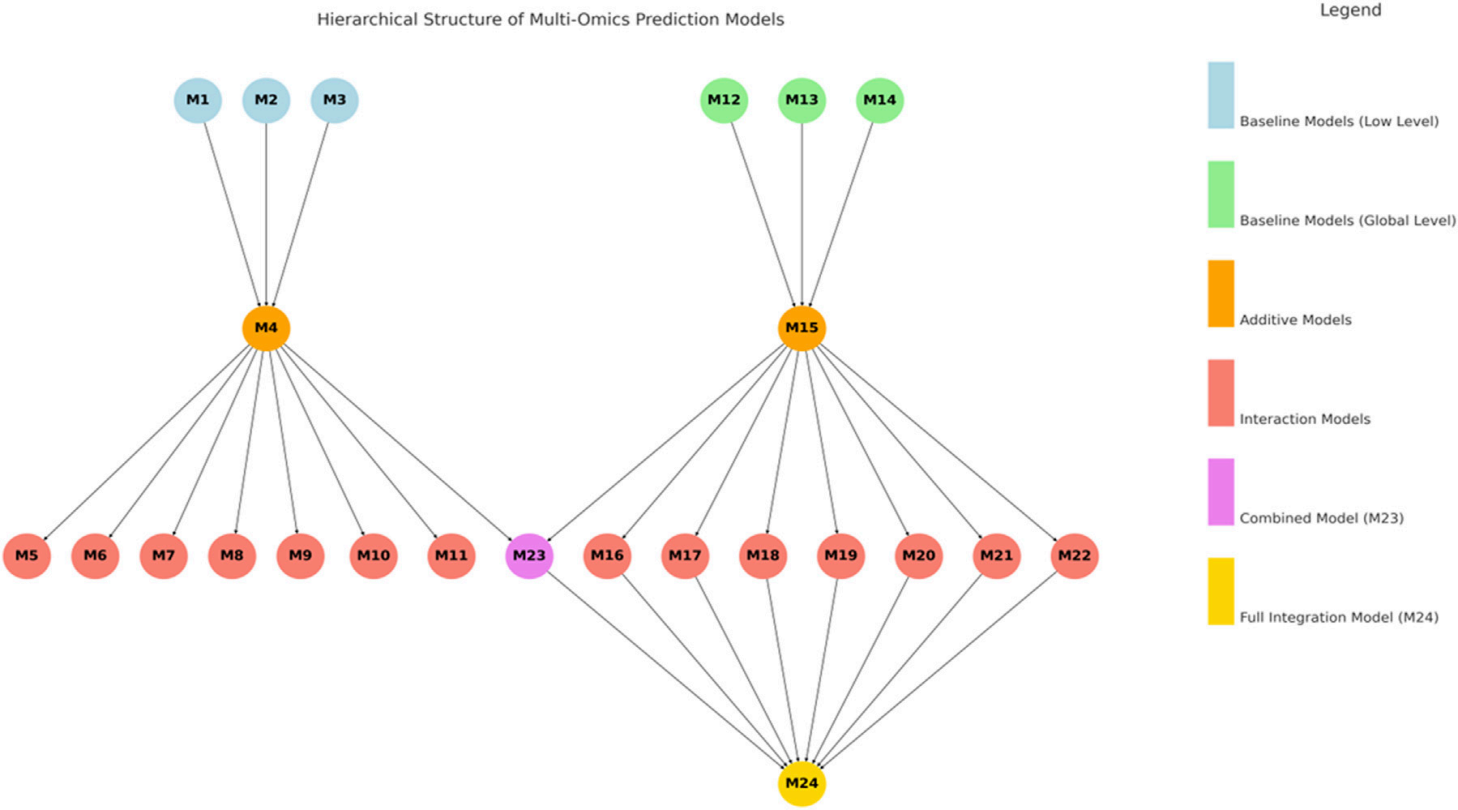
Hierarchical structure of multi-omics prediction models, displaying low- and global-level additive models and interaction models, along with combined and full integrated models.

The 24 evaluated models represent a structured hierarchy of increasing complexity, ranging from single-omics baselines to fully integrated multi-omics predictors. Models M1 to M3 and M12 to M14 serve as baselines, each incorporating a single omics layer—genomics (g), transcriptomics (t), or metabolomics (m)—at either the low (subscript _L) or global level (subscript _G) (Figure 1). Building upon these, models M4 and M15 combine all three omics layers additively within their respective low or global levels, while M23 incorporates additive terms from both levels without interaction effects. A second tier of models (M5 to M11 and M16 to M22) introduces pairwise interaction terms to capture potential nonlinear and hierarchical relationships among omics layers. These interactions are modeled using both upper and lower triangular matrices resulting of matrix multiplication of two original kernels. Models M5 to M11 extend M4 by adding one to three pairwise interactions at the low level, while M16 to M22 do the same for M15 at the global level. The most comprehensive model, M24, integrates all low- and global-level additive terms with all global-level interaction terms, representing a fully fused multi-omics framework. This systematic design allows for a nuanced assessment of how each omics source and its interactions contribute to improving genomic prediction accuracy.

### Heritability estimates

For each model provided in Table 2, its corresponding variance components were computed using the full datasets. Then, for each model, the variance component of error (Ve) and the genetic variance component (VP) were computed. For the computation of VP, all the variance components contained in each model were summed. For example, for model M4, the variance component VP was computed as $VP=\sigma_{g}^{2}+\sigma_{t}^{2}+\sigma_{m}^{2}$ , while for model M5, VP was computed as $VP=\sigma_{g}^{2}+\sigma_{t}^{2}+\sigma_{m}^{2}+\sigma_{\text{gt}}^{2}+\sigma_{\text{gtp}}^{2}$ , and in a similar fashion, the variance component VP was computed for the remaining models. Then, the heritability of each model was computed as $h^{2}=\frac{VP}{VP+Ve}$ .

### Cross-validation and evaluation metrics

To evaluate and compare the predictive performance of different models, we used the random cross-validation method, where a percentage of samples is randomly selected as a training set and the remaining lines are used as the testing set. In our experiments, we use 50% of the samples as a training set and the remaining 50% as a testing set over 20 partitions. We used a 50/50 split for cross-validation to ensure a balanced and robust estimation of accuracy under a conservative evaluation scenario.

To evaluate prediction performance, we compute the normalized root mean squared error (NRMSE) and the average Pearson correlation (APC) metrics in each of the 20 partitions using testing sets. The average of the NRMSE and APC of these 20 partitions across the traits is reported as prediction accuracy for each dataset.

While both metrics (APC and NRMSE) reflect prediction performance, NRMSE measures the absolute relative prediction error; on the other hand, APC (average Pearson correlation) evaluates the consistency of predictions with observed values regardless of scale, making them complementary. To assess the convergence of the posterior distribution, we used trace plots and verified that the potential scale reduction factor (Gelman–Rubin statistic) was <1.1 across all parameters.

## Results

We present the results in four sections. The first section reports the results for the Rice210 dataset. The second section provides the results for the Maize282 dataset, the third section provides the results for the Maize368 dataset, and the fourth section summarizes the results across datasets.

### Rice210

In Table 3, models M19 and M22 exhibited the highest prediction accuracy in the rice dataset and a high heritability value (0.863), followed closely by model M21 (0.862) and model M20 (0.861). In contrast, model M1 (0.666) showed the lowest heritability. Consequently, models M19 and M22 outperformed M21, M20, and M1 in terms of heritability by 0.116%, 0.232%, and 29.57%, respectively.

**TABLE 3:** Estimates of posterior mean of variance (V_i_) and heritability (h^2^) from the Rice210 dataset across traits.

Model	V_P__mean	V_P__sd	V_e__mean	V_e__sd	h^2^_mean	h^2^_sd
M1	77.062	144.134	38.111	67.687	**0.666**	0.157
M2	95.784	175.781	18.857	33.515	0.809	0.053
M3	70.170	129.160	22.433	40.478	0.727	0.077
M4	71.497	131.506	15.801	28.059	0.793	0.055
M5	69.712	128.361	16.469	29.456	0.787	0.053
M6	70.676	129.974	16.622	29.675	0.788	0.048
M7	67.688	124.616	17.036	30.379	0.781	0.062
M8	70.488	129.982	17.112	30.677	0.781	0.049
M9	68.211	125.988	17.418	31.139	0.777	0.063
M10	68.372	126.246	17.435	31.118	0.776	0.060
M11	68.027	125.776	17.675	31.607	0.772	0.059
M12	109.774	202.336	25.023	44.225	0.806	0.063
M13	111.953	204.362	20.790	37.559	0.833	0.025
M14	93.087	170.306	20.608	37.657	0.813	0.036
M15	92.597	169.404	16.565	30.055	0.847	0.017
M16	103.214	188.912	17.103	30.995	0.857	0.020
M17	102.799	187.758	17.070	30.930	0.857	0.017
M18	102.493	187.731	17.034	30.897	0.856	0.019
M19	109.274	199.995	17.170	31.097	**0.863**	0.020
M20	106.963	195.456	17.282	31.335	0.861	0.018
M21	107.491	196.457	17.111	30.996	0.862	0.018
M22	110.724	202.461	17.593	31.875	**0.863**	0.019
M23	78.856	144.249	15.238	27.357	0.828	0.032
M24	89.822	164.262	15.928	28.503	0.838	0.037

Bold values denotes the worst and best estimates of heritability.

**FIGURE 2:**
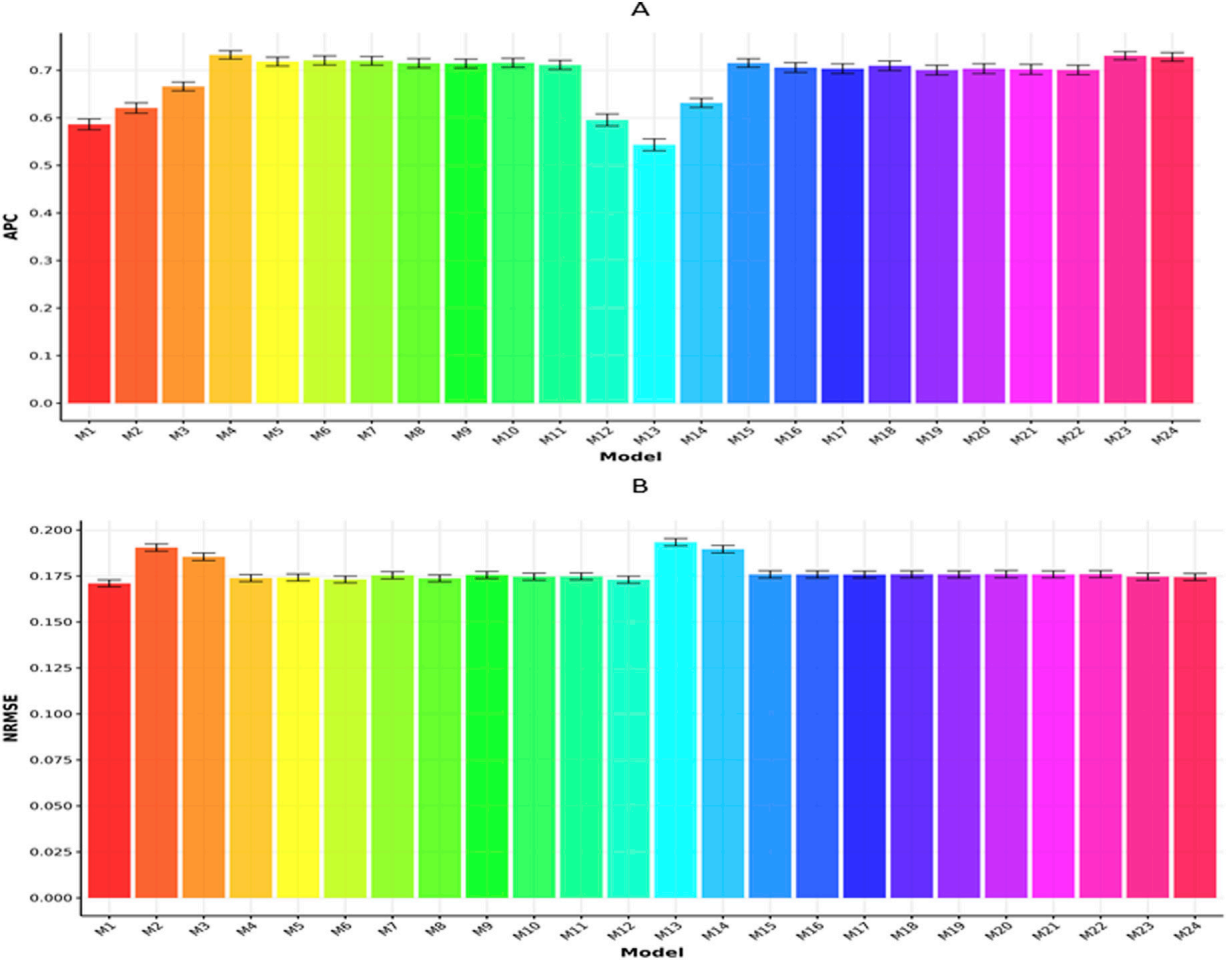
Prediction performance of each model in terms of APC **(A)** and NRMSE **(B)** for the Rice210 dataset. Error bars represent standard deviations across 20-fold cross-validation.

In Figures 2A,B, we present the results for the Rice210 dataset in terms of APC and NRMSE, respectively. In Figure 2A, we can observe that model M4 exhibited the highest APC value (0.7324 ± 0.008), followed closely by model M23 (0.7306 ± 0.0086) and model M24 (0.7281 ± 0.0090). In contrast, model M13 (0.5434 ± 0.0125) showed the lowest APC. Consequently, model M4 outperformed M23, M24, and M13 in terms of APC by 0.246%, 0.591%, and 34.781%, respectively.

Model M12 = G (genomics) using genomic-only data produced an APC of 0.5956 ± 0.0125, while model M23 = G (genomics) + T (transcriptomics) + M (metabolomics), which integrates all three omics layers, achieved an APC of (0.7324 ± 0.008). This represents a 22.96% improvement in prediction accuracy due to omics integration.

However, in Figure 2B, we can observe that model M4 exhibited the lowest NRMSE value (0.1003 ± 0.0016), followed closely by model M23 (0.1021 ± 0.0016) and model M5 (0.1022 ± 0.0017). In contrast, model M13 (0.1247 ± 0.0017) showed the largest NRMSE. Consequently, model M4 outperformed M23, M5, and M13 in terms of NRMSE by 1.795%, 1.894%, and 24.327%, respectively.

### Maize282

As shown in Table 4, model M17 attained the highest heritability estimate (0.807), with model M19 (0.804) and models M21 and M22 (both 0.803) yielding slightly lower values. In contrast, model M2 (0.345) recorded the lowest heritability. Accordingly, model M17 surpassed M19, M21, M22, and M2 in heritability by 0.373%, 0.498%, 0.498%, and 133.913%, respectively.

**TABLE 4:** Estimates of posterior mean of variance components (V_i_) and heritability (h^2^) from the Maize282 dataset across traits.

Model	V_g__mean	V_g__sd	V_e__mean	V_e__sd	h^2^_mean	h^2^_sd
M1	382.202	1173.784	172.264	464.831	0.659	0.108
M2	294.031	979.809	413.711	1227.466	**0.345**	0.060
M3	345.915	1145.114	329.699	937.095	0.456	0.123
M4	440.775	1361.122	152.962	408.773	0.710	0.081
M5	435.008	1349.434	167.915	446.892	0.694	0.090
M6	426.439	1300.493	175.455	478.378	0.685	0.088
M7	443.654	1370.434	157.978	421.567	0.706	0.089
M8	431.281	1331.394	181.369	489.226	0.674	0.091
M9	433.742	1347.032	172.508	458.596	0.688	0.097
M10	431.039	1335.374	177.673	477.820	0.677	0.097
M11	429.684	1342.524	184.911	489.197	0.669	0.099
M12	624.124	1936.033	159.934	441.158	0.769	0.077
M13	414.492	1377.521	440.686	1338.788	0.400	0.073
M14	495.751	1668.184	349.142	1006.293	0.520	0.136
M15	685.346	2125.390	147.142	407.136	0.799	0.056
M16	707.529	2192.624	147.820	407.395	0.800	0.059
M17	724.953	2246.408	145.278	399.245	**0.807**	0.056
M18	701.319	2175.069	147.014	408.095	0.801	0.058
M19	731.771	2271.649	147.658	405.264	0.804	0.060
M20	721.242	2249.651	148.925	409.034	0.799	0.068
M21	735.898	2292.289	147.627	407.976	0.803	0.062
M22	735.365	2277.802	148.204	410.638	0.803	0.063
M23	529.615	1616.155	146.115	396.228	0.757	0.063
M24	556.607	1708.231	148.529	397.585	0.758	0.067

Bold values denotes the worst and best estimates of heritability.

**FIGURE 3:**
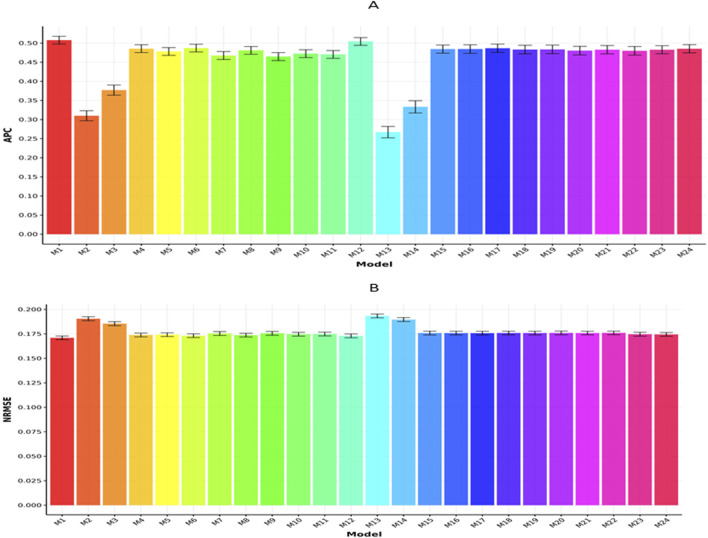
Prediction performance of each model in terms of APC **(A)** and NRMSE **(B)** for the Maize282 dataset. Error bars represent standard deviations across 20-fold cross-validation.

Figures 3A,B display the outcomes for the Maize282 dataset based on APC and NRMSE metrics, respectively. As illustrated in Figure 3A, model M1 achieved the highest APC score (0.5078 ± 0.0101), with model M12 (0.5043 ± 0.0101) and model M24 (0.4854 ± 0.0108) ranking next. In contrast, model M13 registered the lowest APC value (0.267 ± 0.0150). As a result, M1 exceeded the APC performance of M12, M24, and M13 by 0.694%, 4.615%, and 90.187%, respectively.

Meanwhile, Figure 3B shows that model M1 obtained the lowest NRMSE (0.1710 ± 0.0019), indicating superior predictive accuracy. It was followed by model M12 (0.1730 ± 0.0019) and model M6 (0.1732 ± 0.0019 ). Conversely, model M13 had the highest NRMSE (0.1934 ± 0.0020 ). Therefore, M1 outperformed M12, M6, and M13 in terms of NRMSE by 1.169%, 1.287%, and 13.99%, respectively.

### Maize368

Table 5 indicates that models M16 and M19 achieved the highest heritability value (0.829). Models M20 and M22 followed closely with estimates of 0.826, while M15 and M17 (both 0.823) ranked third with slightly lower values. In contrast, model M3 exhibited the lowest heritability (0.290). Thus, M16 and M19 exceeded the heritability of M20, M22, M15, M17, and M3 by 0.363%, 0.363%, 0.729%, 0.729%, and 197.132%, respectively.

**TABLE 5:** Estimates of posterior mean of variance components (V_i_) and heritability (h^2^) from the Maize368 dataset across traits.

Model	V_g__mean	V_g__sd	V_e__mean	Ve_sd	h^2^_mean	h^2^_sd
M1	25.115	67.569	8.272	22.646	0.705	0.077
M2	33.408	93.164	10.490	27.391	0.690	0.094
M3	10.145	27.096	29.906	82.120	**0.279**	0.064
M4	27.451	75.048	7.664	20.231	0.739	0.069
M5	26.877	73.222	8.760	23.650	0.719	0.068
M6	27.001	73.651	7.998	21.042	0.722	0.072
M7	26.673	72.784	7.835	20.838	0.731	0.067
M8	26.233	71.617	9.163	24.584	0.704	0.076
M9	26.211	71.367	8.768	23.623	0.712	0.071
M10	26.469	72.127	8.170	21.786	0.719	0.071
M11	25.913	70.622	8.926	23.626	0.700	0.078
M12	39.334	106.403	7.500	20.228	0.811	0.047
M13	42.967	119.407	9.641	25.235	0.765	0.073
M14	16.888	44.869	27.743	76.357	0.395	0.097
M15	40.514	110.253	7.263	19.444	0.823	0.040
M16	45.076	123.978	7.273	19.243	**0.829**	0.043
M17	41.274	112.465	7.385	19.601	0.823	0.046
M18	40.740	110.509	7.479	20.008	0.821	0.042
M19	44.416	120.970	7.369	19.688	**0.829**	0.045
M20	43.728	119.316	7.502	20.043	0.826	0.049
M21	41.589	112.550	7.414	19.834	0.821	0.047
M22	43.660	118.309	7.521	19.939	0.826	0.046
M23	31.901	86.917	7.331	19.406	0.782	0.048
M24	33.442	91.384	7.731	20.507	0.779	0.059

Bold values denotes the worst and best estimates of heritability.

**FIGURE 4:**
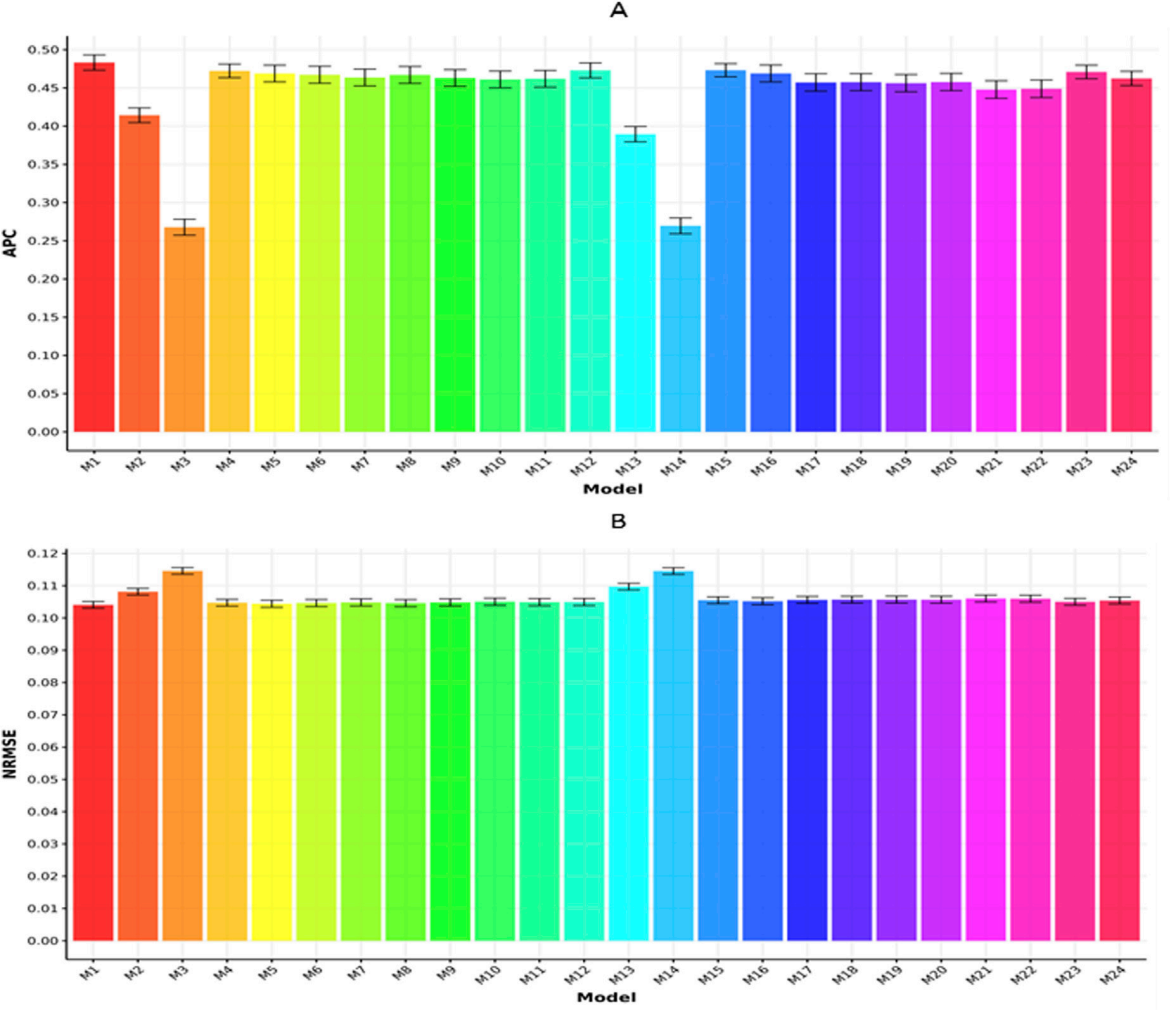
Prediction performance of each model in terms of APC **(A)** and NRMSE **(B)** for the Maize368 dataset. Error bars represent standard deviations across 20-fold cross-validation.

Figures 4A,B summarize the results for the Maize368 dataset with respect to APC and NRMSE, respectively. In Figure 4A, model M1 recorded the highest APC value (0.4832 ± 0.0100), followed by models M15 (0.4732 ± 0.0086) and M12 (0.473 ± 0.0098). On the other hand, model M3 showed the lowest APC (0.2678 ± 0.0105). Accordingly, M1 surpassed the APC values of M15, M12, and M3 by 2.113%, 2.156%, and 80.433%, respectively.

In Figure 4B, model M1 reported the minimum NRMSE (0.1041 ± 0.0100), reflecting the most accurate predictions. This was closely followed by model M5 (0.1044 ± 0.0011) and models M6 and M8, both registering an NRMSE of 0.1046 ± 0.0011. In contrast, model M3 exhibited the highest NRMSE (0.1146 ± 0.0010). Consequently, M1 outperformed M5, M6, M8, and M3 in NRMSE by 0.288%, 0.480%, 0.480%, and 10.087%, respectively.

### Across dataset

According to Table 6, across all traits and datasets, model M19 exhibited the highest heritability value (0.832). This was closely followed by model M22 (0.831), while models M16, M17, M20, and M21 (each with 0.829) showed marginally lower estimates. Conversely, model M3 demonstrated the lowest heritability (0.487). Consequently, M19 exceeded the heritability of M22, M16, M17, M20, M21, and M3 by 0.160%, 0.402%, 0.402%, 0.402%, 0.402%, and 70.725%, respectively. Overall, across all datasets, we observed that the most complex models—specifically those excluding the simpler predictors used in models M1 to M3 and M12 to M14—consistently reported higher heritability estimates. This suggests that integrating the three sources of information (genomic, metabolic, and transcriptomic data) enhances the ability to capture meaningful biological signals rather than noise.

**TABLE 6:** Estimates of posterior mean of (V_i_) and heritability (h^2^) across traits and datasets.

Model	V_g__mean	V_g__sd	V_e__mean	V_e__sd	h^2^_mean	h^2^_sd
M1	161.460	461.829	72.882	185.055	0.677	0.114
M2	141.074	416.251	147.686	429.457	0.615	0.069
M3	142.077	433.790	127.346	353.231	**0.487**	0.088
M4	179.908	522.559	58.809	152.354	0.747	0.068
M5	177.199	517.006	64.381	166.666	0.733	0.070
M6	174.705	501.373	66.692	176.365	0.732	0.069
M7	179.338	522.611	60.950	157.595	0.739	0.073
M8	176.001	510.998	69.215	181.496	0.720	0.072
M9	176.055	514.796	66.231	171.119	0.726	0.077
M10	175.293	511.249	67.759	176.908	0.724	0.076
M11	174.541	512.974	70.504	181.477	0.714	0.079
M12	257.744	748.257	64.152	168.537	0.795	0.062
M13	189.804	567.097	157.039	467.194	0.666	0.057
M14	201.909	627.786	132.498	373.436	0.576	0.090
M15	272.819	801.682	56.990	152.212	0.823	0.038
M16	285.273	835.171	57.399	152.544	0.829	0.041
M17	289.675	848.877	56.578	149.925	0.829	0.040
M18	281.517	824.436	57.176	153.000	0.826	0.040
M19	295.154	864.205	57.399	152.016	**0.832**	0.042
M20	290.644	854.808	57.903	153.471	0.829	0.045
M21	294.993	867.099	57.384	152.935	0.829	0.042
M22	296.583	866.191	57.773	154.151	0.831	0.043
M23	213.457	615.774	56.228	147.664	0.789	0.048
M24	226.624	654.626	57.396	148.865	0.792	0.054

Bold values denotes the worst and best estimates of heritability.

**FIGURE 5:**
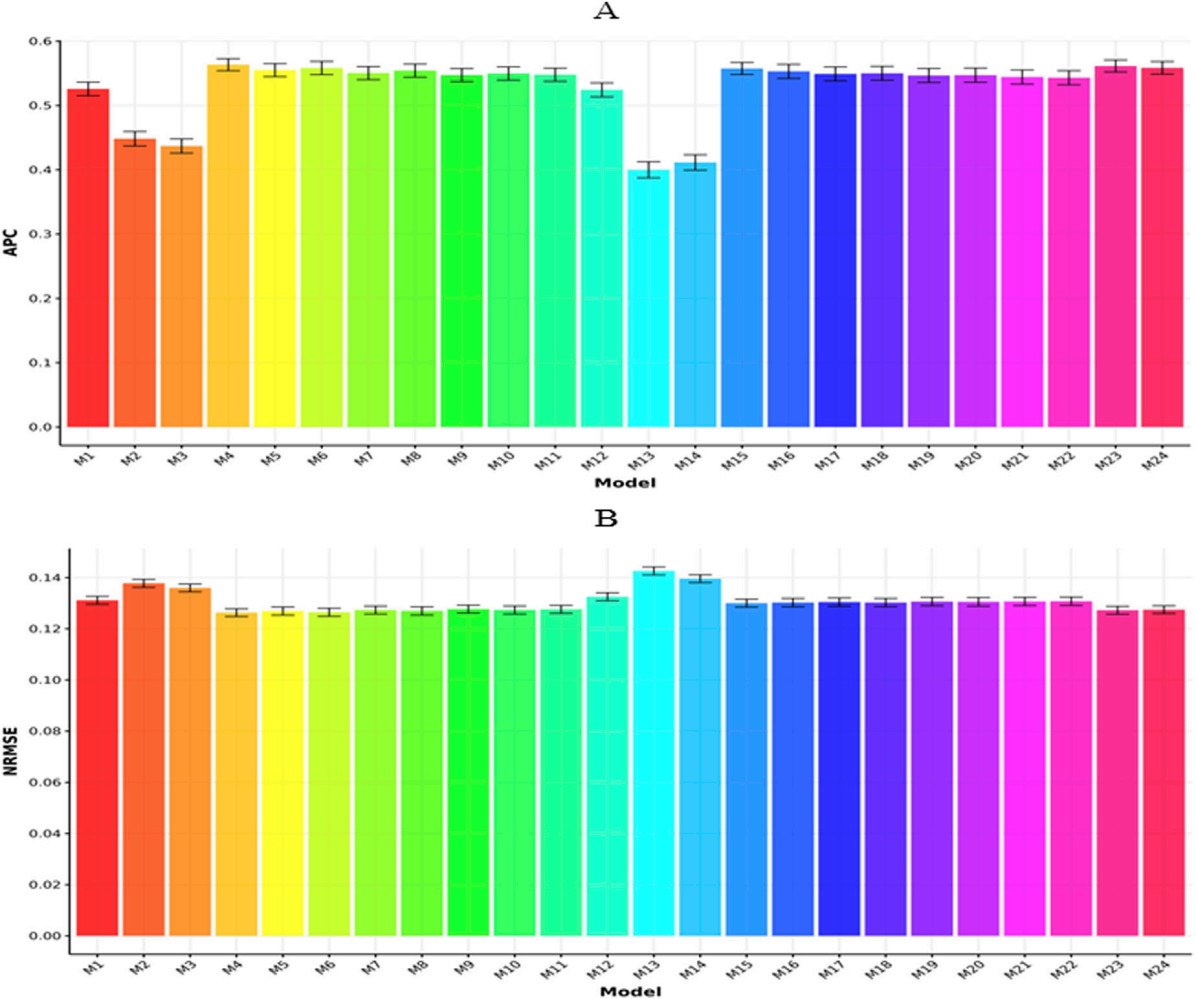
Prediction performance of each model in terms of APC **(A)** and NRMSE **(B)** for across datasets. Error bars represent standard deviations across 20-fold cross-validation.

Figures 5A,B present a comparative analysis across datasets based on APC and NRMSE, respectively. As illustrated in Figure 5A, model M4 achieved the top APC value (0.5634 ± 0.0094), closely followed by models M23 (0.5614 ± 0.0094) and M24 (0.5586 ± 0.0097). In contrast, model M13 registered the lowest APC score (0.3999 ± 0.0125). Thus, M4 outperformed M23, M24, and M13 in APC by margins of 0.356%, 0.859%, and 40.885%, respectively.

In terms of NRMSE (Figure 5B), model M4 yielded the lowest value (0.1263 ± 0.0015), indicating the highest level of predictive precision. Model M6 (0.1265 ± 0.0016) and model M5 (0.1269 ± 0.0016) followed in performance. Conversely, model M13 showed the greatest NRMSE (0.1426 ± 0.0016). Accordingly, M4 exhibited superior performance over M6, M5, and M13 by 0.158%, 0.475%, and 12.91%, respectively.

### Summary of results

The study assessed 24 statistical models integrating genomics, transcriptomics, and metabolomics across three datasets—Rice210, Maize282, and Maize368—to determine their effectiveness in enhancing genomic prediction accuracy.

Our results support the idea that integrating multi-omics data into genomic prediction models has the potential to improve predictive accuracy by leveraging complementary biological information. However, it is hindered by significant differences in dimensionality, measurement scales, noise levels, and missing data patterns. Addressing these issues through advanced preprocessing, normalization, regularization, and modeling strategies is essential for the effective and unbiased integration of multi-omics datasets.

#### Heritability estimates

Models that integrated all three omics layers (e.g., M19 and M22) generally showed the highest heritability across datasets. Rice210: M19 and M22 had the highest heritability (0.863). Maize282: M17 was highest (0.807). Maize368: M16 and M19 reached 0.829. The lowest heritability was often found in single-omics models, particularly those using only metabolomics (e.g., M3).

#### Prediction accuracy

**FIGURE 6:**
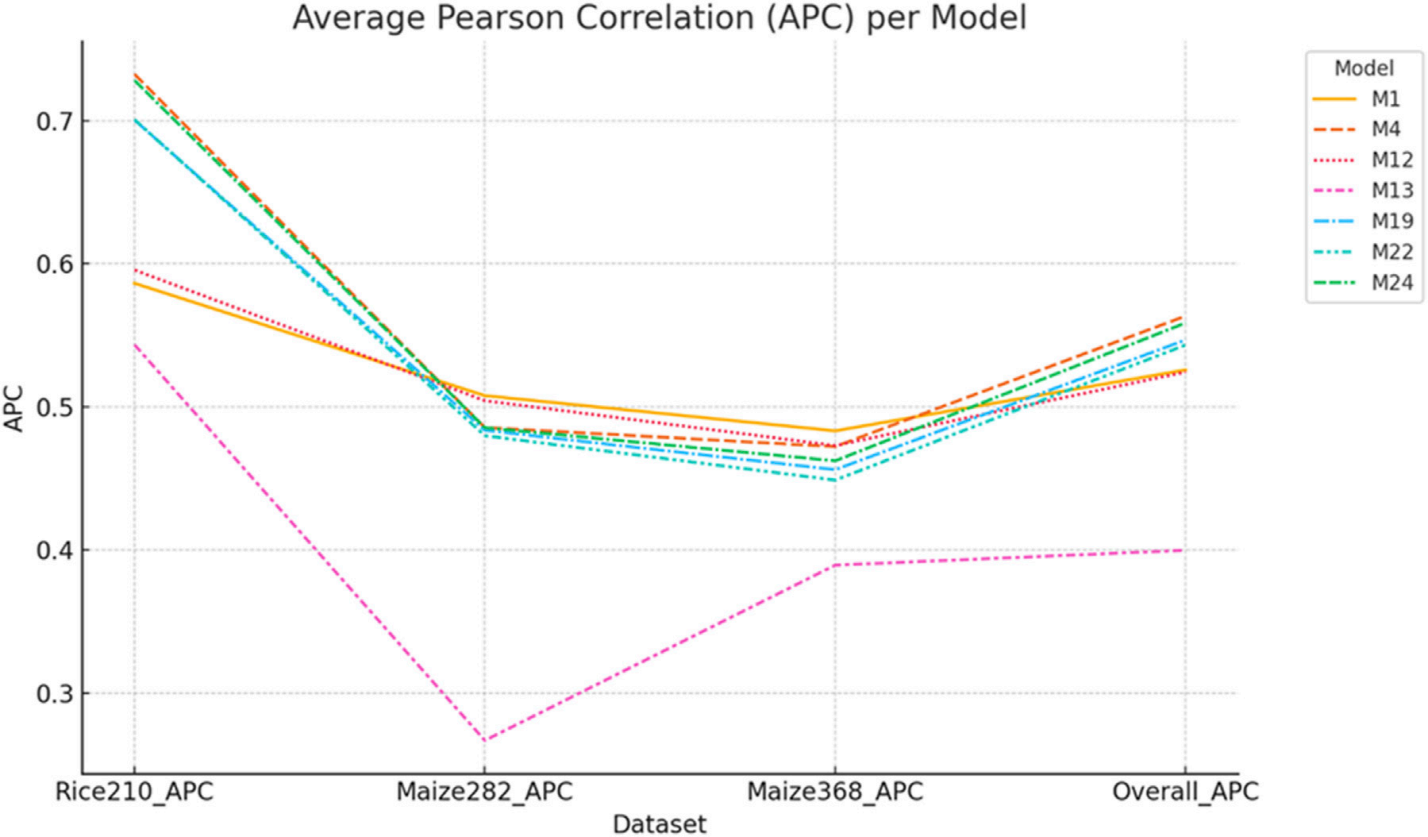
APC values of selected models across Rice210, Maize282, Maize368, and overall datasets. Models M4 and M24 showed balanced performance, while M1 excelled in maize but not rice. M13 (transcriptomics with the Gaussian kernel) had the lowest APC across all datasets. Error bars represent standard deviations across 20-fold cross-validation.

**FIGURE 7:**
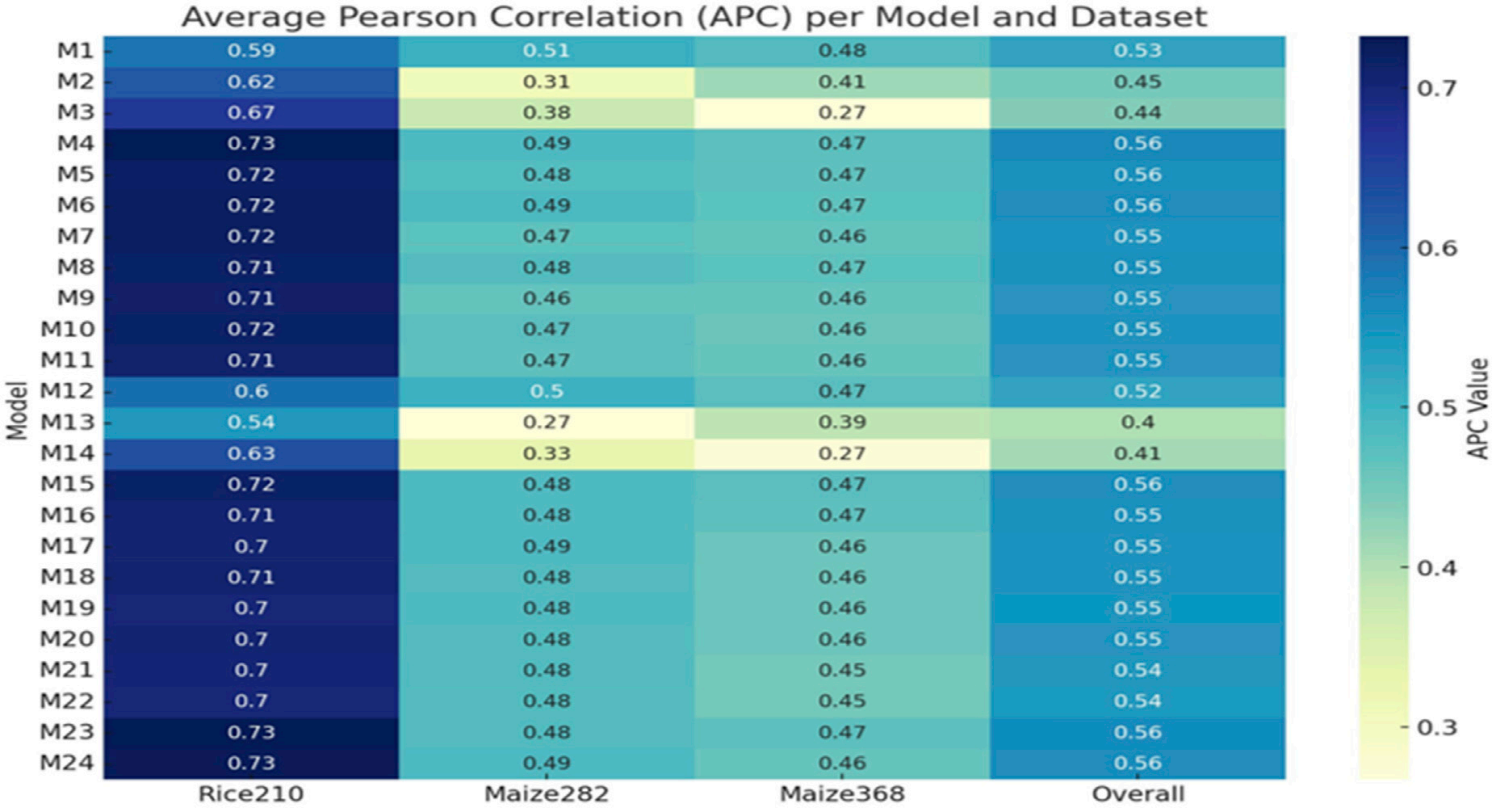
Heatmap of APC values across all models (M1–M24) and datasets (Rice210, Maize282, Maize368, and overall).

For the Rice210 dataset, the best-performing (Figures 6, 7) was M4 (additive integration of *g*_*L*_ + *t*_*L*_ + *m*_*L*_), which achieved the highest Pearson correlation (APC: 0.7324) and the lowest NRMSE (0.1003).

For the Maize282 (Figures 6, 7), the best model was M1 (only genomic info, linear kernel), which surprisingly outperformed multi-omics models in prediction accuracy (APC: 0.5078, NRMSE: 0.1710). For the Maize368 (Figures 6, 7), the best model was again M1, achieving the highest prediction accuracy (APC: 0.4832, NRMSE: 0.1041). In terms of cross-dataset trends, while multi-omics models improved heritability across all datasets, improvements in prediction accuracy were dataset-specific. Model M4, which used only additive effects from the three omics layers (no interactions), consistently performed well across datasets. Gaussian kernel models (e.g., M13) generally underperformed compared to linear kernel-based models.

#### Implications

Integrating multi-omics data has shown promise in improving heritability estimation, particularly for complex traits. However, increases in heritability do not always translate into improved prediction accuracy, underscoring the importance of careful model selection and data preprocessing. Although sophisticated models incorporating interaction terms (e.g., M19 and M22) effectively capture biological variance, they do not consistently enhance predictive performance. This discrepancy suggests the possibility of heritability overestimation or model overfitting. Therefore, although our framework for multi-omics integration is promising, further research is necessary to refine the approach and achieve optimal predictive utility.

Integrating genomics, transcriptomics, and metabolomics is a promising strategy for enriching genomic prediction models, particularly in understanding trait architecture.

Nevertheless, the choice of integration strategy, data type, and model complexity critically affects performance. The study supports the continued development of multi-omics frameworks, balancing complexity with predictive utility.

## Discussion

Despite the promise of GS in accelerating genetic gain in plant breeding programs, its widespread adoption in real-world applications remains constrained by several challenges. Key among these is the variability in prediction accuracy across traits, environments, and populations, often limiting the robustness and generalizability of the models (Crossa et al., 2017; Hickey et al., 2017). Moreover, the complex genetic architecture of many agronomic traits—often governed by numerous small-effect loci and subject to genotype-by-environment interactions—poses a significant barrier to achieving consistent and high predictive performance. Additionally, limitations in training population size, data quality, and the inability to fully capture the underlying biology of traits further impede the effective application of GS in breeding pipelines (Xu et al., 2017).

Our results align with recent developments in omics-based genomic prediction. Several studies have shown that combining genomics with transcriptomic or metabolomic data improves trait prediction, particularly when trait heritability is moderate or low. Compared with traditional models like GBLUP or kernel methods, deep learning architectures—especially those utilizing multi-layer feedforward or convolutional layers—can better capture nonlinear patterns inherent in omics data. The consistent superiority of MLP-based models in our results aligns with the findings of Zingaretti et al. (2020) and Montesinos-López et al. (2021).”

In response to these limitations, a variety of methodological strategies have been explored to improve the performance and reliability of GS. Among the most promising is the integration of multi-omics data, including genomics, transcriptomics, and metabolomics, which offer complementary layers of biological information. These integrative approaches aim to enhance the predictability and interpretability of GS models by capturing intermediate phenotypes and regulatory mechanisms that mediate the genotype-to-phenotype relationship (Fernie and Schauer, 2009; Acharjee et al., 2016). For example, transcriptomic data can provide insights into gene expression patterns linked to trait variability, while metabolomic profiles may reflect physiological states more directly related to phenotype expression. Such integrative models have shown potential to improve the biological relevance of predictions and increase their accuracy across different contexts (Azodi et al., 2020).

The integration of omics data into genomic prediction frameworks is thus emerging as a critical frontier for enhancing predictive accuracy and model interpretability. By leveraging synergistic information from genomics and other omics layers, researchers can better account for the biological complexity of traits, ultimately leading to more precise selection decisions. This approach aligns with systems biology paradigms, which emphasize the interconnected nature of biological data and advocate for a holistic perspective in predictive modeling (Sandhu et al., 2022). Moreover, integrating omics data enables breeders to gain mechanistic insights into trait architecture, supporting both prediction and discovery and offering a dual benefit to crop improvement programs.

Interestingly, the performance gain from integrating omics varied by dataset. In rice, where metabolomic and transcriptomic signals are rich and traits show complex regulation, omics integration yielded substantial gains. In contrast, in the chickpea dataset, where transcriptomic features may have less variance relative to genomic information, the improvement was more modest. These differences illustrate the context dependency of multi-omics modeling, highlighting that data type relevance varies by species and trait architecture.”

Using only genomic information (model M1) led to relatively lower prediction accuracy compared to integrated models (M19–M22), especially in datasets with high-dimensional transcriptomics. This highlights the added value of capturing gene expression patterns or metabolite activity, which can serve as proximal indicators of phenotypic variance. Our study reinforces the view that multi-omics layers provide complementary biological information not captured by markers alone.

Our empirical results underscore the value of an integrative approach to genomic prediction. By incorporating transcriptomic and metabolomic data along with genomic information, we observed a notable increase in heritability across all three datasets. However, a significant improvement in prediction accuracy was observed in only one dataset. This gain in accuracy is not merely incremental—it offers compelling empirical evidence for the critical importance of multi-omics integration within genomic prediction frameworks. These findings highlight the advantages of expanding the data foundation of GS models beyond genomics alone and support the systematic inclusion of all available omics data when feasible. As plant breeding moves further into a data-driven era, leveraging the full spectrum of biological information is no longer optional but essential for achieving the next generation of genetic gain.

Integrating genomic, transcriptomic, and metabolomic data did not, however, enhance prediction accuracy in two of the datasets, thus indicating that multi-omics integration is not universally advantageous. This result highlights key statistical challenges inherent to combining heterogeneous data types. Omics datasets often differ in scale, dimensionality, noise levels, and correlation structures, making integration complex. These differences can complicate model training, particularly in high-dimensional settings where the number of features vastly exceeds the number of observations, increasing the risk of overfitting and reducing generalizability. Additionally, the contribution of each omics layer may vary across samples, with some data sources offering useful signals in certain cases while adding noise in others. Such variability underscores the need for careful preprocessing, feature selection, and model design to fully harness the potential of multi-omics approaches.

Furthermore, the presence of multicollinearity within and across omics layers can inflate variance estimates and obscure the identification of truly informative predictors. Another critical issue is the potential for redundant or weakly informative signals in additional omics layers, which may dilute the predictive power when not appropriately weighted or regularized. The statistical challenge of determining which features contribute meaningfully to the prediction—and how to combine them effectively—requires sophisticated modeling strategies such as dimension reduction, penalization, or multi-view learning approaches.

These findings underscore the need for rigorous and statistical frameworks that can manage the complexity and high dimensionality of multi-omics data while also capturing their complementary information. Addressing these challenges is essential to fully harness the promise of integrative omics in genomic prediction and avoid misleading conclusions based on suboptimal integration methods.

In general, despite the demonstrated efficiency of the integration of multi-omics data in prediction, there is still considerable room for improvement in learning from these different datasets. For this reason, the integration of genomics, transcriptomics, and metabolomics represents a transformative strategy to overcome current limitations in genomic prediction. Our findings highlight the empirical and theoretical advantages of this approach and provide strong justification for future breeding programs to adopt multi-omics data integration as a core component of predictive breeding methodologies.

### Additional considerations

Although the current study provides a comprehensive comparison of multi-omics integration strategies for genomic prediction, several limitations merit attention. First, the lack of *post hoc* biological interpretation of model outputs limits our understanding of why certain omics layers contributed more effectively in specific datasets. For example, the consistent outperformance of simple genomic models in the maize datasets suggests that not all omics layers contributed meaningful biological signals, yet this observation remains unexplored. Second, although the final remarks highlight the importance of model interpretability tools such as SHAP values (Shapley additive explanations help us understand how complex models make their predictions), these were not implemented in the current study. Incorporating such tools in future analyses could enhance biological insights and practical usability by breeders. Finally, all datasets used in this study are derived from single-environment trials, which restricts the generalizability of findings to real-world breeding conditions that are inherently multi-environmental and subject to genotype-by-environment interactions. As a result, future work should prioritize model validation under diverse environmental scenarios and consider integrating G × E effects into multi-omics prediction frameworks. Addressing these limitations will be essential for the successful translation of multi-omics prediction models into practical breeding applications.

### Final remarks

Although the results are promising, some limitations must be acknowledged. First, all datasets were collected under a single-environment condition, limiting our ability to evaluate genotype-by-environment interactions. Second, the early fusion strategy may not fully exploit hierarchical interactions between omics types. Future work should explore advanced integration methods (e.g., deep learning methods with attention mechanisms or graph neural networks) and test model robustness across environments and larger populations. Additionally, evaluating trait-specific model performance would provide more refined insights.

Given the increasing availability of large-scale public omics databases and computational resources, there is a strong incentive to build flexible, modular platforms that can be customized by crop, trait, and available data type. Open-source, community-driven initiatives are likely to accelerate the adoption of multi-omics genomic prediction in diverse agricultural contexts.

Additionally, the importance of explainable models cannot be overstated. As multi-omics models grow in complexity, understanding which features or interactions are driving predictions becomes essential for both interpretability and acceptance by breeders. Model explainability tools such as SHAP values or saliency maps in deep learning can aid in identifying the biological significance of predictors (Lundberg and Lee, 2017). Applying such methods to the current modeling framework may offer breeders mechanistic insights that support not just selection but hypothesis generation and discovery. Moreover, the integration of proteomic and epigenomic data remains an untapped opportunity not only in the context of genomic prediction (Wang et al., 2024b) but also in other fields like human medicine (Lin et al., 2025) and animal science (Wang et al., 2024a). While this study focused on genomics, transcriptomics, and metabolomics, recent advances in high-throughput proteomics and epigenetic profiling (e.g., DNA methylation and histone modifications) have shown potential to capture trait-associated regulatory variation that is not accessible through other omics layers (Langfelder and Horvath, 2017; Chen et al., 2021). Including these additional layers may further enhance both the biological insights and predictive performance of multi-omics models.

Another aspect not extensively discussed in the current manuscript is the role of tissue specificity and developmental timing in transcriptomic and metabolomic data acquisition. Omics layers are inherently dynamic; transcript abundance and metabolite levels change throughout development and in response to environmental stimuli. As such, integrating omics data collected at a single time point or from a limited tissue type might obscure important regulatory mechanisms relevant to trait expression (Rai et al., 2021; Do et al., 2020). Future research should consider the temporal and spatial aspects of omics data collection to improve predictive resolution.

## Conclusion

The integration of genomics, transcriptomics, and metabolomics into genomic prediction models has shown promise in enhancing predictive accuracy, although improvements have been observed only in certain datasets compared to models relying solely on genomic data. This variability underscores both the potential and the complexity of incorporating multi-omics information into prediction frameworks. Although empirical evidence highlights the added value of multi-omics integration, it also reveals significant challenges in achieving effective data fusion and optimal model performance. Nonetheless, the incorporation of multi-omics data holds great potential to enhance predictive power and enable more informed decision-making in plant breeding programs. These findings emphasize the transformative capacity of holistic, data-driven strategies in modern breeding efforts. We strongly advocate for continued research aimed at developing and refining integrative multi-omics frameworks. Their effective implementation could substantially improve the identification of superior candidate lines and accelerate genetic gain in genomic selection programs.

## Data Availability

Publicly available datasets were analyzed in this study. These data can be found at: https://doi.org/10.6084/m9.figshare.19312205.v1.
